# Perspectives on HIV partner notification, partner HIV self‐testing and partner home‐based HIV testing by pregnant and postpartum women in antenatal settings: a qualitative analysis in Malawi and Zambia

**DOI:** 10.1002/jia2.25293

**Published:** 2019-07-19

**Authors:** Rebecca B Hershow, Chifundo C Zimba, Oliver Mweemba, Kasapo F Chibwe, Twambilile Phanga, Wezzie Dunda, Tulani Matenga, Wilbroad Mutale, Benjamin H Chi, Nora E Rosenberg, Suzanne Maman

**Affiliations:** ^1^ Department of Health Behavior University of North Carolina at Chapel Hill Gillings School of Global Public Health Chapel Hill NC USA; ^2^ University of North Carolina Project Malawi Lilongwe Malawi; ^3^ Department of Health Promotion and Education University of Zambia School of Public Health Lusaka Zambia; ^4^ Department of Health Policy School of Public Health University of Zambia Lusaka Zambia; ^5^ Department of Obstetrics and Gynecology University of North Carolina at Chapel Hill School of Medicine Chapel Hill NC USA

**Keywords:** HIV testing, male partner engagement, prevention of mother‐to‐child transmission, Malawi, Zambia, qualitative research

## Abstract

**Introduction:**

HIV testing male partners of pregnant and postpartum women can lead to improved health outcomes for women, partners and infants. However, in sub‐Saharan Africa, few male partners get HIV tested during their partner's pregnancy in spite of several promising approaches to increase partner testing uptake. We assessed stakeholders’ views and preferences of partner notification, home‐based testing and secondary distribution of self‐test kits to understand whether offering choices for partner HIV testing may increase acceptability.

**Methods:**

Interviewers conducted semi‐structured interviews with HIV‐negative (N = 39) and HIV‐positive (N = 41) pregnant/postpartum women, male partners of HIV‐negative (N = 14) and HIV‐positive (N = 14) pregnant/postpartum women, healthcare workers (N = 19) and policymakers (N = 16) in Malawi and Zambia. Interviews covered views of each partner testing approach and preferred approaches; healthcare workers were also asked about perceptions of a choice‐based approach. Interviews were transcribed, translated and analysed to compare perspectives across country and participant types.

**Results:**

Most participants within each stakeholder group considered all three partner testing strategies acceptable. Relationship conflict was discussed as a potential adverse consequence for each approach. For partner notification, additional barriers included women losing letters, being fearful to give partners letters, being unable to read and men refusing to come to the clinic. For home‐based testing, additional barriers included lack of privacy or confidentiality and fear of experiencing community‐level HIV stigma. For HIV self‐test kits, additional barriers included lack of counselling, false results and poor linkage to care. Preferred male partner testing options varied. Participants preferred partner notification due to their respect for clinical authority, home‐based testing due to their desire to prioritize convenience and clinical authority, and self‐test kits due to their desire to prioritize confidentiality. Less than half of couples interviewed selected the same preferred male partner testing option as their partner. Most healthcare workers felt the choice‐based approach would be acceptable and feasible, but noted implementation challenges in personnel, resources or space.

**Conclusions:**

Most stakeholders considered different approaches to partner HIV testing to be acceptable, but concerns were raised about each. A choice‐based approach may allow women to select their preferred method of partner testing; however, implementation challenges need to be addressed.

**Abbreviations**
HTC: HIV testing and counselling
IDI: in‐depth interview
IPV: intimate partner violence
PMTCT: prevention of mother‐to‐child transmission
SSA: sub‐Saharan Africa
UTH: University Teaching Hospital
WHO: World Health Organization

## 1 Introduction

Despite advances in HIV prevention of mother‐to‐child transmission (PMTCT) in sub‐Saharan Africa (SSA), pregnant and postpartum women face a high risk of HIV acquisition from infected male partners and subsequent mother‐to‐child transmission 1, 2, 3, 4, 5. Similarly, many HIV‐infected women have male partners who are also HIV‐infected and do not know their HIV status 6, 7. Engaging male partners of pregnant and postpartum women in HIV testing can be a first key step towards improved health outcomes for HIV‐positive or HIV‐negative women, their male partners, and their infants, including better uptake of and adherence to antiretroviral treatment and reduced HIV‐related morbidity and mortality 6, 7, 8, 9, 10, 11, 12. However, in SSA, less than one‐third of male partners of pregnant women report testing for HIV 10, 13, 14, 15, 16 and this represents an important gap in current programmes. Barriers include poor access to HIV testing services, gender norms and relationship dynamics 17, 18.

Several evidence‐based practices increase male partner HIV testing in antenatal settings in SSA, including partner notification 19, 20, home‐based testing 21, 22, 23 and secondary distribution of self‐test kits 24, 25, 26, 27. These approaches target male partners of HIV‐positive and HIV‐negative pregnant and postpartum women. In a partner notification approach that has been highly effective in our setting, healthcare workers provide pregnant women with an invitation for their male partner to attend the health facility for important pregnancy information. For partners who do not return, healthcare workers actively trace the partners by phone or home visit to encourage HIV testing 20. In home‐based testing, healthcare workers visit women and their male partners in their home and offer HIV testing and counselling services. Finally, with secondary distribution of HIV self‐test kits, women are trained to use self‐test kits, bring self‐test kits home and instruct their partner in the testing procedures 28. HIV test results are reported back to healthcare workers and those with positive results are referred for care 28.

Despite strong evidence behind these approaches and endorsement by the World Health Organization (WHO) 12, 28, no single strategy has consistently achieved the ambitious 90% HIV testing target set by the Joint United Nations Commission on HIV/AIDS. Given the challenges inherent to male partner HIV testing, a “one‐size‐fits‐all” approach – with any HIV testing modality – may face limited success. Offering women options for partner HIV testing may improve uptake, since women may have a better understanding of which testing modality would work best for them and their partner. To the best of our knowledge, however, such a “choice‐based approach” has never been evaluated and comparative views about different partner HIV testing practices among stakeholders are not well understood. In this study, we aim to assess among a range of stakeholders in Malawi and Zambia: (1) the perceived acceptability and preferences of three different male partner HIV testing modalities; and (2) the perceived acceptability of a choice‐based approach for male partner HIV testing in antenatal settings.

## 2 Methods

### 2.1 Recruitment

This qualitative, formative study was conducted in Lilongwe, Malawi and Lusaka, Zambia. Potential participants included: (1) pregnant/postpartum women stratified by HIV status; (2) male partners of these index pregnant/postpartum women; (3) healthcare workers; and (4) policymakers.

Individuals were recruited from a variety of settings using purposive sampling; the research team's long‐standing presence in Malawi and Zambia informed the selection of recruitment sites. In Malawi, we recruited pregnant/postpartum women from Bwaila District Hospital in Lilongwe, a district maternity hospital operated by the Malawi Ministry of Health. In Zambia, pregnant/postpartum women were recruited from University Teaching Hospital (UTH) and Kamwala Health Centre. Pregnant/postpartum women enrolled in the study were asked to invite their male partners to participate if they were interested in doing so. Healthcare workers, including community health workers, nurses, counsellors, midwives and health educators, were recruited from the above‐mentioned facilities, all of which provide HIV prevention services. Policymakers focused on PMTCT and HIV testing were recruited from the Ministry of Health, implementing partners, and donor agencies in each country.

### 2.2 Data collection

From June 2017 to May 2018, one‐on‐one qualitative in‐depth interviews (IDIs) were conducted by five trained interviewers in private rooms. Prior to data collection, interviewers reviewed the translated guides to ensure they understood the meaning and purpose of the questions. Sample size was designed for each stakeholder group in each country to reach saturation 29. Eligible participants provided written informed consent before participation; if participants had low literacy, a witness confirmed that the individual understood before signing. Interviews lasted approximately 60 minutes and were conducted in English, Chichewa, Nyanja or Bemba, depending on the participant's preference.

Interviewers used semi‐structured interview guides tailored to participant type. Interviews covered a range of strategies to improve HIV prevention, care and treatment services. For this analysis, we focused on male partner HIV testing modalities, which were asked about midway through the interviews. Prior to asking about each participant's hypothetical views, each strategy was described consistently across interviews, including for HIV‐positive and HIV‐negative women. The description of partner notification was that male partners would receive a letter from the clinic informing them of their HIV risk and inviting them to the clinic for HIV testing; if a male partner did not visit, the clinic staff would call him to encourage testing. We recognize that asking HIV‐negative women about partner notification deviated from the WHO definition, which specified that partner notification should target partners of HIV‐positive women 28. However, we feel it is a missed opportunity not to engage male partners of HIV‐negative women with partner notification in antenatal settings. We believe woman's testing, regardless of her result, is an important motivator for male partner testing 30. Including male partners of HIV‐negative women in partner notification efforts is particularly important in high prevalence settings such as Malawi and Zambia. The description of home‐based HIV testing was that community health workers would visit the home to offer HIV testing to male partners. The description of secondary distribution of HIV self‐test kits was that women would be provided with the HIV self‐test kits and training on how to use them; then, the women would distribute the HIV self‐test kits to their male partners. For all approaches, interviewers emphasized that the woman's consent would be required before approaching her male partner. The interviewer corrected misunderstandings. All participants received a small transport allowance.

### 2.3 Data analysis

Audio recordings were translated and transcribed into English. A central codebook was developed with input from both country teams. Deductive codes were derived from interview guides about each major topic to index the interviews by topics 31. Coding was conducted by country‐specific teams in NVivo12 (QSR International, Doncaster, Australia). To ensure researchers were applying codes consistently, both coding teams coded the same transcripts, discussed discrepancies and revised the codebook. Code reports were reviewed and code summaries were written for country summary reports to assess overarching patterns in views on each male partner testing approach 32. To facilitate a deeper analysis, matrices were developed to systematically compare relevant responses across participants and countries 33. Similarities and differences in findings by stakeholder group and/or country were identified and described and illustrative quotes were selected.

### 2.4 Ethics approvals

Our protocol was approved by the University of North Carolina at Chapel Hill Institutional Review Board (Chapel Hill, NC, USA), the University of Zambia Biomedical Research Ethics Committee (Lusaka, Zambia), and the Malawi National Health Sciences Research Committee (Lilongwe, Malawi).

## 3 Results

### 3.1 Sociodemographic characteristics of women and male partners

Overall, 143 participants were interviewed (Table 1): 80 pregnant/postpartum women, 28 of their male partners, 19 healthcare workers and 16 policymakers. Women were younger than male partners and most were married (Table 2). All male partners reported past HIV testing. Of the 28 participating couples, 13 were both HIV‐negative, nine were both HIV‐positive and two were HIV sero‐discordant with HIV‐positive women.

**Table 1:** Number and percentage of study participants by participant type and country^a^

	Pregnant or postpartum women N (%)		Male partners N (%)		Healthcare workers N (%)	Policymakers N (%)	Total N (%)
	HIV‐negative	HIV‐positive	Female partner HIV‐negative	Female partner HIV‐positive			
Malawi	20 (51)	20 (49)	7 (50)	8 (57)	10 (53)	10 (63)	75 (52)
Zambia	19 (49)	21 (51)	7 (50)	6 (43)	9 (47)	6 (38)	68 (48)
Total	39 (100)	41 (100)	14 (100)	14 (100)	19 (100)	16 (100)	143 (100)

a Percentages may not sum to 100 due to rounding.

**Table 2:** Sociodemographic characteristics of pregnant/postpartum women and male partners in Malawi and Zambia (N = 108)^a^

	HIV‐positive women (N = 41)	Male partners of HIV‐positive women^b^ (N = 14)	HIV‐negative women (N = 39)	Male partners of HIV‐negative women (N = 14)
	N (%) or mean			
Age in years (mean)	29	37	26	31
Highest education level completed				
None	16 (39)	4 (31)	9 (23)	0 (0)
Primary school	17 (41)	5 (38)	13 (33)	6 (43)
Secondary school	6 (15)	2 (15)	8 (21)	2 (14)
Tertiary education	2 (5)	2 (15)	9 (23)	6 (43)
Marital status				
Married	40 (98)	13 (100)	36 (92)	14 (100)
Single	0 (0)	0 (0)	3 (8)	0 (0)
Separated/divorced	1 (2)	0 (0)	0 (0)	0 (0)
Employment status				
Employed (full‐ or part‐time)	20 (49)	12 (92)	15 (38)	13 (93)
Homemaker	16 (39)	0 (0)	18 (46)	0 (0)
Student	0 (0)	0 (0)	2 (5)	0 (0)
Unemployed/other	5 (12)	1 (8)	4 (10)	1 (7)
Male partner's HIV testing history				
Has ever tested	34 (83)	14 (100)	37 (95)	14 (100)
Never tested	3 (7)	0 (0)	2 (5)	0 (0)
Don't know	4 (10)	0 (0)	0 (0)	0 (0)
HIV status				
Negative	0 (0)	2 (15)	39 (100)	13 (93)
Positive	41 (100)	9 (69)	0 (0)	0 (0)
Unknown	0 (0)	2 (15)	0 (0)	1 (7)

^a^Percentages may not sum to 100 due to rounding; ^b^Missing data (except for male partner's HIV testing history): Male partners of HIV‐positive women: N = 1.

### 3.2 Views on HIV partner notification

Most women and male partners thought partner notification was acceptable, explaining that male partners would find the letter and tracing attempt motivating because they would take seriously the healthcare provider recommendation to undergo HIV testing. “There needs to be someone to shake them [men] up. ‘You need to know your status!’ So, the phone method is good, you send letters to them…they cannot refuse, they can accept” (Male partner of HIV‐negative woman, Malawi). Some emphasized that it was a viable option because women could explain the process to their partners before presenting the letter.

Many women and male partners identified challenges. Some explained that relationship conflict could be an adverse consequence. Specifically, they believed some men might suspect their wife was involved in arranging the letter or call and feel their wife violated their privacy or did not trust them. Others thought the approach would be ineffective, since both letters and calls are easy to evade. Some HIV‐negative women in Malawi pointed out that the female partner's HIV status may influence the male partner's response. A few said that men may be more likely to test if their partner was HIV‐positive; others said that men may feel offended that they were asked to test if their partner was HIV‐negative.

Most healthcare workers in Malawi and Zambia and policymakers in Malawi agreed that partner notification was acceptable, describing similar interventions found to be effective. Some, especially policymakers in Zambia, noted the implementation challenges involved with partner notification: women losing letters, being fearful to give partners letters, being unable to read, and letters not motivating men to test. Additionally, these participants expressed mixed feelings about follow‐up tracing. Some thought it would be helpful for providers to talk to men over the phone. Others noted that phone calls could be expensive, that some men may be hard to reach by phone, and that some men may react poorly if they find the approach overly aggressive.

Overall, participants agreed that partner notification would be an effective approach for some men, but not others. Stakeholders felt that some male partners would feel motivated by the partner notification approach, while others may feel offended and/or avoid testing.

### 3.3 Views on home‐based HIV testing

The majority of women and male partners thought home‐based testing was acceptable, convenient, and would provide savings in time and transport money. Furthermore, they liked that community health workers would be present to provide counselling. They also believed the home‐based visit would show that community health workers care about them. Many explained that it would be important for women to schedule the visit or discuss it with their partners in advance to avoid surprising them.

Some women and male partners also expressed concerns. A few felt that it would be difficult to schedule home visits since men are often at work and may feel pressured or offended by an unannounced home visit. If men felt offended, some warned that conflict within the relationship, including violence, could ensue. Some, particularly HIV‐positive pregnant/postpartum women, emphasized concerns around confidentiality, noting that neighbours might assume they are HIV‐positive, leading to stigma and discrimination. One participant explained: “When you start following us home, you find that maybe a neighbour had been followed before and so will know there's nothing else, they are here for HIV testing” (HIV‐positive woman, Zambia). Participants noted that these concerns were more prominent in densely populated areas.

Most healthcare workers and policymakers found home‐based testing to be acceptable. They explained that home‐based testing would make HIV testing more accessible, since men often prefer to avoid clinic settings and the associated transportation costs. In Zambia, some healthcare workers and policymakers added that home‐based testing has been successfully implemented. “Home visits are good, because when calling someone, if you give someone a letter or you call him by phone, that they will come, they won't come. But if you follow them they will be forced ‘just test me’ because you have followed him” (Healthcare worker, Zambia). These healthcare workers and policymakers had mixed feelings about whether home‐based visits would compromise confidentiality. Some thought it might provide more privacy, comfort, and confidentiality; others felt that they may experience stigma and discrimination from neighbours if they learned about the purpose of the visit. A few stated that stigma surrounding home‐based visits could be addressed through community‐level education to normalize and promote home‐based visits, and provision of additional bundled health services so they were not singularly associated with HIV testing.

A few healthcare workers and policymakers highlighted concerns. Healthcare workers in Malawi explained that men may refuse the home‐based visit if they feel it is aggressive or a privacy violation. Healthcare workers in Zambia explained that women with male partners who are married to other women would not be reachable. Policymakers in Malawi explained that home‐based testing is too expensive and potentially stigmatising; and policymakers in Zambia explained that home visits would be difficult to schedule since men are often out of the house and there may be privacy issues if children are home.

Overall, all stakeholders thought accessibility and savings in time and transport money for clients were benefits and privacy violations and stigma were potential risks of home‐based HIV testing.

### 3.4 Views on secondary distribution of HIV self‐test kits

The majority of women and male partners felt secondary distribution of HIV self‐test kits was acceptable. The approach was thought to be convenient, ensured confidentiality and allowed men to avoid the clinic. Some also said that they liked that secondary distribution of self‐test kits would allow them to do couples testing alone. Many emphasized the importance of high‐quality training so that women would be able to introduce self‐test kits to their partner, train their partner to administer the HIV test, and accurately interpret the test result. In Malawi, many women liked that secondary distribution of self‐test kits would allow them to play a large role in facilitating the testing process. Similarly, many male partners in Malawi, especially partners of HIV‐negative women, liked how secondary distribution of self‐test kits would allow women to teach them how to test. Initially, one male partner reacted to secondary distribution of self‐test kits negatively, explaining that men may not trust women to adequately administer the test. “I would say that the challenge would be belittling the woman saying, ‘Ah no, you cannot instruct me like that!’ Many would react in this manner” (Male partner of HIV‐negative woman, Malawi). After the interviewer clarified that the women can teach their male partners to test themselves, he changed his mind and said that this was a good option.

Some pregnant/postpartum women and male partners expressed concerns that individuals would face difficulties administering the test, reading the test results, reporting the results, and seeking treatment if diagnosed with HIV. Others noted that the lack of professional counselling may lead to self‐harm or poor linkage to treatment. Some explained that secondary distribution of self‐test kits could lead to relationship conflict:

…According to my own understanding I think it [secondary distribution of self‐test kits] can bring disagreements because a man is a difficult person. You find that we test and she finds that I'm not well. Instead of me accepting, you find that I'll start accusing…Meaning that it will be difficult for peace to prevail in homes. (Male partner of HIV‐positive woman, Zambia)

These participants were concerned that men would associate their female partner's request for HIV self‐test kits with distrust or suspicions of promiscuous behaviour.

Some healthcare workers and policymakers felt that secondary distribution of self‐test kits was acceptable, noting that it would make HIV testing services easier to access and could help ensure confidentiality. Other healthcare workers and policymakers, however, expressed concerns about implementation challenges, including performance of the HIV self‐test kits (and the possibility of false negative results), difficulties around monitoring and distributing self‐test kits, and high cost for HIV self‐test supplies:

Maybe they [other HIV testing approaches] don't offer the same convenience as an oral self‐test kit, but they also don't have the risk that somebody is going to test right there in the house and say I am negative, I don't need to go for treatment, when they are actually positive. (Policymaker, Malawi)

Others expressed that the lack of professional counselling could lead people to avoid reporting the results and seeking treatment. They also expressed concerns about relationship conflict, especially for individuals who receive a positive result and sero‐discordant couples.

Overall, we found that there was higher acceptability for secondary distribution of HIV self‐test kits among pregnant/postpartum women and male partners as compared to healthcare workers and policymakers in both Malawi and Zambia.

### 3.5 Most preferred male partner testing modality

All women and male partners were asked about their preferred male partner HIV testing modality and responses varied (Table 3). While male partners were split in their preferences for the three partner testing modalities, preferences differed between HIV‐negative and HIV‐positive women. Stated preferences were often guided by participants’ views on perceived advantages and disadvantages of each approach, as described above. Most HIV‐positive women selected partner notification and secondary distribution of self‐test kits, often citing concerns around confidentiality and HIV stigma with home‐based testing. Most HIV‐negative women in Malawi selected partner notification and home‐based testing, commonly mentioning issues around false or misinterpreted results and lack of counselling with secondary distribution of self‐test kits. When comparing preferred male partner testing modalities within couples, less than half of HIV‐negative and HIV‐positive pregnant/postpartum women and their partners chose the same preferred male partner testing option.

**Table 3:** Pregnant/postpartum women's and male partners’ perception of most preferred male partner HIV testing modality^a^

	Partner notification	Home‐based testing	Secondary distribution of self‐test kits
Malawi			
HIV‐negative women (N = 20)	8 (40)	9 (45)	3 (15)
HIV‐positive women (N = 20)	8 (40)	3 (15)	10 (50)
Male partners of HIV‐negative women (N = 7)	3 (43)	3 (43)	1 (14)
Male partners of HIV‐positive women (N = 8)	3 (38)	2 (25)	3 (38)
Zambia			
HIV‐negative women (N = 19)	3 (16)	5 (26)	11 (58)
HIV‐positive women (N = 21)	9 (43)	3 (14)	8 (38)
Male partners of HIV‐negative women (N = 7)	2 (29)	3 (43)	2 (29)
Male partners of HIV‐positive women (N = 6)	1 (17)	4 (67)	2 (33)
Total			
HIV‐negative women (N = 39)	11 (28)	14 (36)	14 (36)
HIV‐positive women (N = 41)	17 (41)	6 (15)	18 (44)
Male partners of HIV‐negative women (N = 14)	5 (36)	6 (43)	3 (21)
Male partners of HIV‐positive women (N = 14)	4 (29)	6 (43)	5 (36)

a Numbers may not sum to equal the total number of participants and percentages may not sum to equal 100 as some participants selected more than one testing modality and some participants had no preference.

### 3.6 Views on choice‐based approach

When asked about their views on the choice‐based approach for male partner HIV testing, most healthcare workers felt it was acceptable and feasible to implement at the facility‐level. Many explained that the choice‐based approach would engage women in the process and ensure the optimal partner testing option was selected. “But in actual sense it's the woman who knows the husband better. So, it's better we give them the choices” (Healthcare worker, Zambia). Some healthcare workers also identified challenges related to lack of personnel or resources. A few emphasized the need to re‐structure the space and/or services to accommodate male partners. One healthcare worker in Malawi noted that “our health system is often [biased towards] women” and recommended bundling male‐focused health services with HIV testing to attract men. Issues related to couple dynamics were also mentioned, such as women being unable to instruct men to test as they are not the heads of the household, women with male partners who are married to other women facing difficulties, and men continuing to refuse to test. No important differences by country were observed.

## 4 Discussion

To the best of our knowledge, this is the first study to explore perspectives and preferences of three male partner HIV testing strategies. The broad engagement of different stakeholder populations across two countries resulted in a variety of perspectives on acceptability and preferences of the three partner HIV testing approaches. The majority of participants accepted all three partner testing modalities, though – when asked to select their preferred approach – the most common choices differed between groups. Additionally, participants raised perceived barriers, which also varied by target population.

When asked about the three HIV testing modalities, concerns were raised by all stakeholders about potential social harms, most commonly relationship conflict and stigmatization. Most trials, however, evaluating antenatal partner testing approaches found few or no social harms 11, 14, 20, 21, 22, 24, 25, 26, 27, 34, 35. One exception was a passive partner notification evaluation that found the majority of intimate partner violence (IPV) was reported among couples who had received a positive HIV result during couples testing (4/6) 19. HIV disclosure without support has been linked to IPV among pregnant and postpartum women 36, 37, 38, suggesting that more support for HIV‐positive women is needed to ensure a safe disclosure process 19.

Stakeholders identified potential implementation challenges for each HIV testing modality. Although evaluation trials have demonstrated the effectiveness of these male partner HIV testing approaches in antenatal settings 21, 22, 27, 35, 39, 40, 41, implementation research with cost‐effectiveness analysis would be helpful to guide countries when considering partner testing approaches. While cost‐effectiveness studies in the general adult population have been promising 41, 42, 43, 44, evidence of cost‐effectiveness in antenatal settings is still needed.

Preferences around partner HIV testing options varied according to individual priorities, including respect for clinical authority, convenience, and privacy. Some participants preferred the partner notification approach described in the IDI due to respect for clinical authority, noting the importance of receiving a letter directly from healthcare workers and receiving professional HIV counselling. Some preferred home‐based testing as they prioritized convenience and clinical authority, expressing that home‐based testing was highly accessible for clients and linked them to professional HIV counselling. Others preferred secondary distribution of self‐test kits as they prioritized privacy, emphasizing that this approach allowed for testing alone or privately with a partner. Although preferences did not substantially differ across most stakeholder groups, HIV‐positive women tended to prefer partner notification and secondary distribution of self‐test kits, while HIV‐negative women tended to prefer partner notification and home‐based testing. Given that HIV‐negative and HIV‐positive women and their male partners have different needs and concerns, offering choices may help a wider range.

Overall, our study provides evidence of acceptability for a choice‐based approach for partner HIV testing. Couples included in the study often did not select the same preferred male partner testing approach, demonstrating a potential disconnect between what pregnant/postpartum women preferred and what men preferred. To address this issue, women may wish to provide the HIV testing choices to their partner before making the decision and should be counselled on the optimal time, place, and way to communicate the testing options. Trainings that address these issues have been found to be effective for secondary distribution of self‐test kits 25 and partner notification 45. Additionally, all stakeholder groups noted the importance of women engaging men around key decisions related to each HIV testing option, such as when to schedule the home visit. It may also be beneficial to provide guidance to pregnant/postpartum women as they consider the HIV testing choices to help them think through which option would be best for their partner.

There are some limitations to the study. The partner testing preferences were hypothetical and should be assessed further in programmatic settings to understand actual preferences and subsequent utilization when provided with choices. Additionally, only one partner notification strategy was explored in the interviews, leaving potential gaps in understanding on the full range of choices. However, acceptability of each element of the approach was assessed separately, facilitating analysis on individual components.

Additionally, there are some limitations to our population. Our sample size is smaller than that in quantitative studies and not selected to be statistically representative. Thus, it is not appropriate for prevalence estimates. However, our sample size was appropriate for qualitative research, as it is aimed at in‐depth understanding of participants’ views 29. Selection bias may be present as female participants may have been more engaged in health services and more inclined to engage their partners than those women who were approached but refused. This is probable as few women were unaware of their male partner's HIV status. Similarly, the men who were successfully recruited to the study may have been more receptive to these interventions and cooperative than the general population. This bias was likely as all male partners knew their HIV status. Furthermore, male partners’ previous methods of HIV testing may have influenced views on male partner testing approaches; we could not explore this in our data. Additionally, the responses of healthcare workers may have been biased by the research team's long‐standing presence in both countries, resulting in potential social desirability.

## 5 Conclusions

We observed a range of preferred male partner HIV testing approaches, suggesting that a choice‐based approach to promote testing among male partners of pregnant/postpartum women could be effective in SSA. Despite high acceptability of such a strategy, there are numerous challenges to consider as the choice‐based approach adds complexity to already burdened health systems. However, challenges should be weighed against the strategy's potential to substantially increase male partner HIV testing by accommodating couples’ differing concerns and needs.

## Competing interests

The authors declare that they have no competing interests.

## Authors’ contributions

All authors have read and approved the final manuscript. B.C., W.M., N.R. and S.M. designed the research study, supported analysis and provided feedback on manuscript drafts. C.Z., O.M., K.C., T.P., W.D. and T.M. contributed to interview guide design, oversaw data collection, analysed the data and provided feedback on manuscript drafts. R.H. analysed the data and wrote the manuscript.
